# Cirrhosis of the Liver

**Published:** 1905-06-17

**Authors:** 


### CIRRHOSIS OF THE LIVER.

In the course of some general remarks upon cirrhosis of the liver Dr. Robert Saundby [1] points out that though unquestionably all cirrhotic livers do not owe their origin to alcoholic poisoning, an immense proportion do so owe it. Some people have suggested that it is not the alcohol itself which is so deleterious to the liver, but some substance associated with it, either as an accidental impurity or one intentionally introduced. Thus Lancereaux credited the baneful effects of excessive consumption of wines to the potassium sulphate used to plaster them, but the disease follows the abuse of such very distinct types of intoxicant that the ordinarily received opinion seems to be the most reasonable. None the less the author is not prepared to deny that micro-organisms may play an important part in determining the lesion, as was suggested by Adami, who claims to have shown that while the bacillus coli communis may be found dead in any section of normal liver, in cirrhotic livers it is present in numbers and full of vitality. Dr. Saundby also believes that the persistent use of pepper and spices, so much affected by Anglo-Indians, may assist the poisonous action of alcohol upon the liver, basing his belief upon experiments of Tinozzi which showed that the addition of pepper or capsicum to the food of dogs and rabbits causes after a certain time a definite increase of hepatic connective tissue.

The author enters a sound warning against the acceptance of a patient's own estimate of his habits as regards alcohol, a warning well justified in the case of the man who formed the text of the paper. This man had always been a great drinker of whisky, consuming as much as a pint a day, but said that since an illness seven or eight years before he had been "moderate," taking no more than on an average six glasses of ale and six of whisky in the day. It is recommended that the best way to arrive at the truth is to ask the patient to give an exact account of an average day of his life, including his ordinary programme of meat and drink, and without laying enough stress upon the question of alcohol to put the patient on his guard. Symptoms are not an accurate index of the anatomical condition of the liver, for an extreme degree of cirrhosis is compatible with good health, and a fatal hæmatemesis may be the first and last symptom of the disease. Slight yellowness of the conjunctiva is a common appearance in cirrhosis of the liver, and depends upon the gastro-duodenal catarrh which is the direct result of the irritation of the mucous membrane by the poison. Paracentesis should be practised as soon as the bulk of the effusion causes distress in respiration or otherwise. Many cases die in spite of everything, but a few recover a degree of health which is remarkable when the probable condition of the liver is taken into account.

Attempts have been made, and are still upon their trial, to establish a collateral circulation with the portal system by effecting adhesions between the viscera and the abdominal parietes. Thus in a considerable number of cases the omentum has been stitched to the anterior abdominal wall, and though the figures are not conclusive, the proceeding appears to hold out some hope of alleviation in cases where the effusion persistently re-collects after tapping.

[1] Practitioner, June 1905.

